# A comparison of dental professionals’ and patients’ perceptions in assessing smile esthetics

**DOI:** 10.1038/s41598-026-48105-1

**Published:** 2026-04-16

**Authors:** Zulal Deniz Guner, Rabia Karaaslan, Ekin Dikici, Fatma Karacaoğlu, Gözde Çobanoğlu, Nilsun Bağış

**Affiliations:** 1 Department of Periodontology, Faculty of Dentistry, Ankara University, Emniyet, Mevlana Blv., No:19/1 Yenimahalle, Ankara, Turkey; 2 Department of Restorative Dentistry, Faculty of Dentistry, Ankara University, Emniyet, Mevlana Blv., No:19/1 Yenimahalle, Ankara, Turkey; 3 Private Clinic, Ankara, Kavaklıdere, John F. Kennedy Cd., No:24/3, Çankaya, Turkey; 4 Ankara University Institute of Health Sciences, Dışkapı Yerleşkesi, Zübeyde Hanım, Şht. Ömer Halisdemir Blv., Ankara, Turkey

**Keywords:** Esthetics, Esthetic perception, Smile, Smile index, Health care, Medical research

## Abstract

Smile aesthetics is one of the primary reasons for patients to seek aesthetic dental treatment. Although various parameters have been proposed to evaluate smile aesthetics, objective and standardised criteria are still under discussion. The aim of this study was to evaluate whether the perception of aesthetic changes differs between dental professionals and laypersons. Frontal photographs of 18 patients were selected from 81 patient photographs. Two full smile images of 18 cases were shown to 86 professionals and 86 layperson participants. Participants were asked to mark the most aesthetic smile and the most important criteria in making their decisions on the prepared forms. Statistical analysis was performed using the Mann-Whitney U test for two-group comparisons and the Kruskal-Wallis H test for comparisons with three or more groups. The rate of choosing the aesthetic smile according to the SI was significantly higher in professionals (*p* < 0.05). In the non-professional group, teeth rate was significantly more often considered the main criterion for aesthetic smile discrimination (*p* < 0.05). The professional group placed greater emphasis on gingival appearance when evaluating smile aesthetics (*p* < 0.05). Aesthetic perception differed between professionals and laypersons. The Smile Index should be interpreted alongside other esthetic parameters and patient expectations.

## Introduction

Facial attractiveness, particularly the smile, plays an important role in an individual’s self-esteem and psychosocial well-being^1–3^. Individuals with aesthetically pleasing smiles are often perceived more positively in social and professional environments^4,5^. However, the perception of smile aesthetics is inherently subjective and influenced by various factors, including age, gender, cultural background, socioeconomic status, and exposure to media and social trends^6,7^. In recent years, increasing aesthetic awareness and the influence of digital media have further raised expectations regarding dental and smile appearance^8^.

Smile aesthetics has therefore become an essential component of contemporary dental treatment planning^9^. Several factors such as tooth position and morphology, gingival display, lip dynamics, and facial harmony contribute to the perception of an attractive smile^10^. Researchers have emphasised various concepts, such as anterior smile line, smile curve, smile index, and dynamic smile symmetry for the evaluation of smile aesthetics^11^. These concepts have been accepted as the primary standard that specialists will rely on in creating an appropriate treatment plan and smile design^12^.

Diverse aesthetic indices and subjective measures, including gingival display, buccal corridors and smile arc have also been utilized to assess smile attractiveness. However, the Smile Index (SI) provides a measurable and objective criterion for evaluating smiles by using the ratio between the intercommissural and interlabial distances^13^.

SI was developed by Ackerman et al. to visually and quantitatively assess the smile. This index is calculated by dividing the intercommissural distance by the interlabial distance during a posed smile^10^. It delineates the region encompassed by the vermilion borders of the lips during a social smile and functions as an effective instrument for comparing smiles either among different individuals or within the same individual over time. Smiling is affected by various factors, including age, gender, personality traits, and emotional state; in this context, lip dynamics are a crucial predictor of aesthetic perception. An aesthetically beautiful smile requires a balanced interplay among the exposed tooth surface, gingival display, and lip movement. Wang et al. reported that SI values typically range from 4.82 to 7.5 in smiles considered aesthetically attractive^14^. Ackerman et al. further noted that lower SI values are associated with a more youthful appearance, and an aesthetically favourable smile generally has a SI greater than 5.0^10^. Despite its simplicity and reliability, the SI has not been widely adopted across aesthetic studies. Furthermore, limited research has examined how SI values genuinely influence smile preference assessment in a clinically relevant way.

Dentists likely have a significant influence on how patients evaluate and judge aesthetics. Previous studies have demonstrated that dental professionals tend to evaluate smiles more critically and with greater emphasis on technical criteria such as tooth alignment, gingival contour, and symmetry. In contrast, laypersons are often more influenced by general impression, tooth brightness, and emotional expression^15^.

Studies typically reveal that dentists hold significantly higher aesthetic standards than patients or non-professionals^16^. However, the dentist’s treatment goals may not always align with the patient’s expectations and desires. On the other hand, aesthetic perception is also influenced by cultural and regional factors. A community’s assessment of an element as aesthetically pleasing may not align with that of another society^17^. Therefore, it is important to learn about patients’ expectations and consider their aesthetic preferences within their sociocultural context prior to initiating treatment.

Based on previous literature, professionals are more likely to judge smiles with appropriate SI values as esthetic. In contrast, laypersons may prioritise subjective indicators such as tooth appearance or whiteness. This study aims to examine the differences in aesthetic judgments between dentists and individuals lacking dental expertise when assessing smiling images categorized by objectively determined SI values. We evaluated aesthetic preferences by comparing pre- and post-orthodontic treatment smiling photographs of the same individuals to determine any variations between the two groups and the nature of these differences.

## Materials and methods

Approval for this study was obtained from the Ankara University Faculty of Dentistry Clinical Research Ethics Committee (36290600/16/2024). All procedures involving human participants were performed in accordance with the principles of the Declaration of Helsinki.

This study initially evaluated 267 patients who had completed maxillary arch expansion with the Invisalign technique. The following inclusion and exclusion criteria were then applied to these patients. Based on these criteria, a total of 81 patients were included in the study.

Inclusion criteria:

- Completion of maxillary arch expansion using the Invisalign technique,
- Availability of standardized pre-treatment and post-treatment intraoral photographs or digital records,
- No missing maxillary anterior teeth,
- Age 18 years or older at the time of treatment.

Exclusion criteria:

- Presence of anterior restorations,
- Presence of any craniofacial anomalies or pathologies,
- Presence of surgical scars resulting from soft tissue procedures, such as cleft lip and/or palate repair,
- History of orthognathic surgery or previous orthodontic treatment, to eliminate potential confounding factors related to major skeletal, dental, or soft tissue alterations that could affect baseline soft tissue dynamics and smile aesthetics.

Pre- and post-treatment photographs of these patients (a total of 162 photos) were employed in the analysis. SI values before and after treatment were calculated by a single examiner using the ImageJ V 1.53 software. To ensure intra-rater reliability, the same examiner repeated the SI measurements of 20 randomly selected patients one month after the initial measurements, under identical conditions. The intraclass correlation coefficient (ICC) was calculated as 0.82, indicating favourable reliability.

The lower limit of the SI was based on the study by Ackerman et al., which reported that young individuals with aesthetic smiles had SI values above 5.0^10^, while the upper limit was established according to Wang et al., who indicated that the aesthetic SI could extend up to approximately 7.5^14^. Among the total pool of 81 patients, 18 patients with one of their pre- or post-treatment SI scores within this range and without major gingival alterations or significant dental crowding that could affect aesthetic perception were included in the evaluation.

Photographs were standardised using consistent lighting (5000 K), camera angles (frontal view at eye level), and resolution (1920 × 1080 pixels) to ensure uniformity across all images. Then, the pre- and post-treatment photographs of these 18 patients were randomly reorganised to minimise recall bias and prepare the evaluation presentation. In total, 86 professionals—including 47 periodontists and 39 dentists from other specialties, all with a minimum of 2 years of clinical experience—and 86 lay participants were recruited. After obtaining written informed consent, evaluators were shown two full smile images of each of the 18 cases side by side. Evaluators were blinded to whether the images were taken before or after treatment. Participants were asked to indicate their aesthetic preference among the presented cases and the factors influencing their choices on a survey form. The evaluation categories concerning the most important aesthetic criteria (teeth, lip, and gingiva) were selected based on commonly reported smile components in previous aesthetic perception studies, which have consistently identified these anatomical components as key determinants in the perception of smile esthetics^16,18^. Each image was displayed for 10 s for evaluation. After completing the evaluation, the participant’s involvement in the study ended. No guidance was provided to the evaluators. The images were clearly projected onto a white screen in a dimly lit room, and each image was shown only once. Participants were also asked to indicate their demographic details, including age, gender, and education level. Although the primary objective of this study was to compare aesthetic perception between dental professionals and laypersons, the education level of lay participants was recorded as an additional variable. Previous studies have suggested that educational attainment may influence aesthetic awareness and perception; therefore, this factor was evaluated to explore potential differences within the layperson group.

### Statistical analysis

Statistical analyses were performed using the SPSS software package (version 22.0; IBM Corp., Armonk, NY, USA). The normality of the data distribution was assessed using the Shapiro–Wilk test. As the data did not meet the assumption of normal distribution, non-parametric tests were applied. The Mann–Whitney U test was employed for comparisons between two independent groups, while the Kruskal–Wallis H test was employed for comparisons involving three or more groups. A *p*-value less than 0.05 was considered statistically significant.

The sample size calculation was performed using G*Power (v3.1.9.4), with an effect size of 0.9 drawn from previous literature reporting large perception differences between dental professionals and laypersons^11^. Based on this effect size, a total of 172 participants (86 per group) was determined to achieve a power of 0.95 at a significance level of 0.05 for two-tailed Mann-Whitney U tests.

## Results

The data of this study, which aimed to compare the perceptions of dentists and patients in evaluating smile aesthetics, were obtained from the records of patients who underwent maxillary arch expansion with the Invisalign technique by an orthodontist in a private clinic and whose treatment was completed.

The age and gender distribution of the participants in the professional (*n* = 86) and non-professional (*n* = 86) groups were similar (*p* > 0.05). While all participants in the professional group had postgraduate education, the education levels of the non-professional group participants were classified within themselves (Tables 1 and 2). A considerable proportion of the layperson group also had postgraduate education.

**Table 1:** Mean age of the study groups.

	Age					
	n	Mean	Median	Minimum	Maximum	sd
**Professionalisation**						
Non Professional	86	25,9	25,0	18,0	36,0	5,0
Professional	86	27,5	27,0	24,0	42,0	2,6
Total	172	26,7	27,0	18,0	42,0	4,0

**Table 2:** Gender and education level distribution of the study population.

	Professionalisation					
	Non Professional		Professional		Total	
	n	%	n	%	n	%
**Gender**						
Male	35	40,7	24	27,9	59	34,3
Female	51	59,3	62	72,1	113	65,7
Total	86	100,0	86	100,0	172	100,0
**Education level**						
Primary School	1	1,2	0	0,0	1	,6
High School	9	10,5	0	0,0	9	5,2
Undergraduate	67	77,9	0	0,0	67	39,0
Postgraduate	9	10,5	86	100,0	95	55,2
Total	86	100,0	86	100,0	172	100,0

Professionals were significantly more likely to select smiles within the aesthetically acceptable SI range (5.0–7.5.0.5) compared with non-professionals (*p* < 0.05). When making aesthetic smile discrimination between the two groups, the non-professional group prioritised the teeth, while the criterion prioritised by the professionals was gingival appearance (*p* < 0.05) (Table 3).

**Table 3:** Comparison of impressive criteria and SI value when discriminating aesthetic smile between professional and non-professional groups.

	Professionalisation						Mann-Whitney U testi		
	n	Mean	Median	Minimum	Maximum	sd	Row Mean	U	p
**SI**									
Non Professional	86	9,0	9,0	6,0	14,0	1,4	70,36	2310	< 0,0001
Professional	86	10,0	10,0	7,0	14,0	1,5	102,64		
Total	172	9,5	9,0	6,0	14,0	1,5			
**Teeth**									
Non Professional	86	12,6	13,0	0,0	18,0	3,4	100,10	2528,5	< 0,0001
Professional	86	11,3	11,0	6,0	18,0	2,6	72,90		
Total	172	11,9	12,0	0,0	18,0	3,1			
**Lip**									
Non Professional	86	3,0	3,0	0,0	11,0	2,2	88,28	3545	0,635
Professional	86	2,9	3,0	0,0	9,0	2,1	84,72		
Total	172	3,0	3,0	0,0	11,0	2,1			
**Gingiva**									
Non Professional	86	2,4	2,0	0,0	15,0	2,5	65,35	1879,5	< 0,0001
Professional	86	3,9	4,0	0,0	8,0	2,0	107,65		
Total	172	3,1	3,0	0,0	15,0	2,4			

When all participants were evaluated to investigate the effect of gender on aesthetic perception, no significant difference was found (*p* > 0.05) (Table 4), but education was found to be effective on aesthetic perception. The rate of individuals with postgraduate education choosing an aesthetic smile within the range of 5–7.5.5 was found to be significantly higher than the other groups. (*p* < 0.05) (Table 5).

**Table 4:**
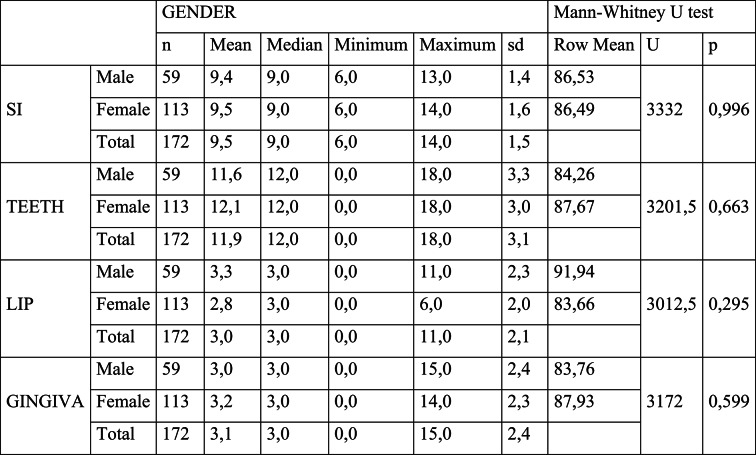
Comparison of SI value and the criteria affecting the discrimination of aesthetic smile between genders.

**Table 5:** Comparison of the SI value and the criteria affecting the discrimination of aesthetic smile between education levels.

Category	Education Level	*n*	Mean	Median	Min	Max	sd	Row Mean	H	*p*	Binary Comparison
SI	1 = High School and Below	10	9,3	9,5	6	11	1,7	88,25	13,4	< 0,0001	3 − 1, 3 − 2
	2 = Undergraduate	67	9	9	6	14	1,4	69,75			
	3 = Postgraduate	95	9,9	10	7	14	1,5	98,13			
	Total	172	9,5	9	6	14	1,5				
Teeth	1 = High School and Below	10	11,7	13	0	16	4,7	95,6	9,6	0,008	2 − 1, 2–3
	2 = Undergraduate	67	12,6	12	0	18	3,3	100,04			
	3 = Postgraduate	95	11,5	11	6	18	2,8	75,99			
	Total	172	11,9	12	0	18	3,1				
Lip	1 = High School and Below	10	3,2	4	0	5	1,9	96,8	0,8	0,685	–
	2 = Undergraduate	67	3,1	3	0	11	2,2	88,35			
	3 = Postgraduate	95	2,9	3	0	9	2,1	84,11			
	Total	172	3	3	0	11	2,1				
Gingiva	1 = High School and Below	10	3,1	2	0	14	4,1	70,65	25,7	< 0,0001	2 − 1, 2–3, 1–3
	2 = Undergraduate	67	2,3	2	0	15	2,3	64,6			
	3 = Postgraduate	95	3,7	4	0	8	2	103,61			
	Total	172	3,1	3	0	15	2,4				

## Discussion

Aesthetics is a primary motivation for patients pursuing dental treatment, and a significant portion of most dental clinical practices are shaped by esthetic dentistry^19^. Creating an aesthetic smile is a subjective art, so it is not possible to define a specific smile type^20–25^.

**Fig. 1:**
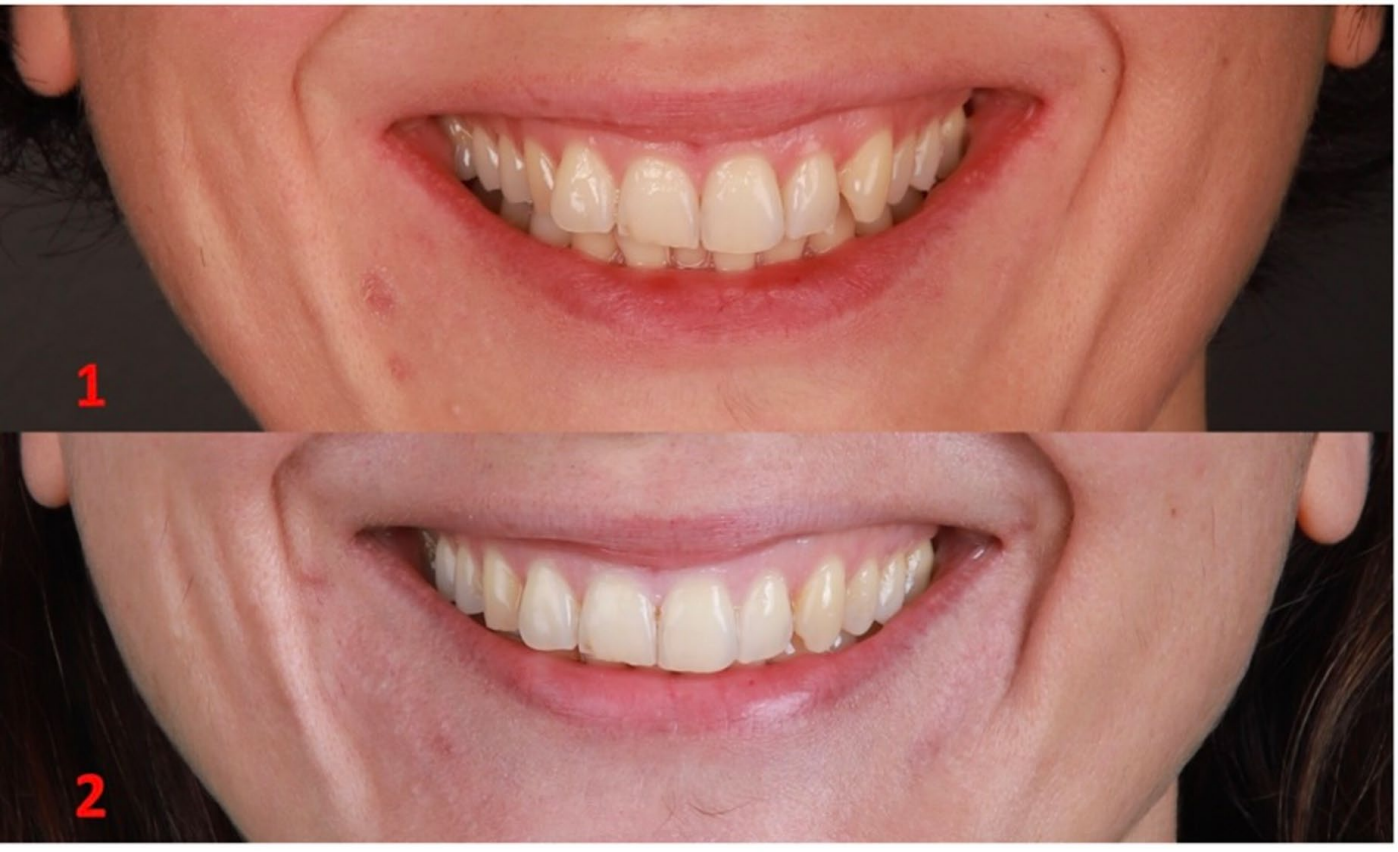
Pre- and post-treatment smile photographs of a patient who underwent maxillary arch expansion using the Invisalign technique. One of the images falls within the esthetically ideal Smile Index (SI) range (5.0–7.5), while the other lies outside this range.

In the literature, various evaluation approaches have been utilized to assess aesthetic perception. To assess the perception of smile esthetics among orthodontists and patients, Machado et al.^26^ presented case photographs to participants in a randomly organized photo album and instructed them to evaluate the photographs using the Visual Analogue Scale (VAS). Similarly, Aldegheishem et al.^27^ utilized VAS to assess the aesthetic perception of both physicians and laypersons. Niknam et al.^28^ examined the attractiveness perceptions of orthodontists, dentists, and laypersons utilizing a Likert scale. In our study, photographs of the same individual with orthodontic treatment and changes in SI, only one of which met the ideal smile criteria, were used for aesthetic evaluation and evaluated by the questionnaire method. The fact that the photographs belonged to the same person eliminated side factors that could affect smile aesthetics. The optional questionnaire questions were designed to eliminate options, presenting aesthetic smiles as a binary yes/no response. Rather than using a continuous scale such as VAS, participants were instructed to select one of two smile photographs and identify the most significant aesthetic criterion from a set of predefined options. This methodology provided a distinct binary option and reduced variability in subjective scaling (Fig. 1).

The patient’s aesthetic perceptions may not align to the standardised protocols and technical practices that affect clinicians’ perspectives^29^. Numerous research studies have assessed the discrepancies in perception between physicians and patients to date^30^. Mehl et al.^31^ applied a questionnaire to assess the difference in aesthetic perception between patients and dentists, comparing pre-treatment and post-treatment photographs of 16 different patients. As a result, they emphasised that there are differences in aesthetic perception between patients and professionals and that it would be useful to take these differences into consideration when planning treatment, to establish good communication and to visualise the treatment results before finalising the treatment. Rodrigues et al.^25^ similarly established that patients’ assessments of smile aesthetics diverged from objective aesthetic standards. In our study, the results regarding the perceptions of smile aesthetics between dentists and patients were assessed. According to the literature, the findings revealed that dentists had a more critical perspective in aesthetic evaluations than patients and assessed the characteristics of an ideal smile with more precision. Conversely, patients’ aesthetic assessments differ based on individual and subjective expectations. Our findings indicate that a smile adhering to ideal or standardized standards may not be perceived as aesthetically pleasing by patients. Therefore, it is essential to consider patient preferences while creating aesthetic smiles and for the clinician to recognize patient expectations regarding treatment efficacy.

Patients and physicians may prioritise different criteria when assessing smile aesthetics. The specialized fields and professional experiences of physicians are variables affecting this difference in perception. Cotrim et al.^18^ assessed the perception of smile aesthetics among three distinct groups: orthodontists, patients and clinicians. They found that orthodontists considered gingival and lip position as the primary criteria, whereas patients and clinicians prioritised features related to tooth shape and alignment.

Our questionnaire survey revealed that professionals placed greater emphasis on gingival components, whereas laypersons focused more on dental appearance. This difference may be partially explained by the professional background of the participants. The professional group in the present study consisted predominantly of periodontists, who are trained to assess soft tissue harmony, gingival contour, and periodontal health in detail. Consequently, their greater sensitivity to gingival display and symmetry may have influenced the overall aesthetic evaluation. Previous studies have also demonstrated that professional training and clinical experience shape aesthetic perception, particularly in relation to soft tissue components of the smile. Therefore, the professional distribution within the sample should be considered when interpreting these findings. Future studies including a more balanced distribution of dental specialties may further clarify the influence of professional background on smile perception.

Our study revealed no significant difference in aesthetic perception between genders (*p* > 0.05). Flores et al.^24^ conducted a study assessing the perception of smile aesthetics and found the following gender distribution: physician participants (*n* = 83; 32 females, 52 males) and lay participants (*n* = 282; 151 females, 131 males). As a consequence, they did not observe any difference in perception between genders. Similarly, Kaya et al.^32^ assessed smile attractiveness across several groups in their study and found no significant gender differences. Nonetheless, certain research in the literature has indicated that women possess higher aesthetic expectations than males when assessing aesthetics in dentistry^26–34^. This study found that gender did not influence aesthetic perception, although similar research indicated that women exhibited greater attentiveness to aesthetics.

Studies have also investigated aesthetic perception among dentists with varying educational backgrounds^16,26,29,35,36^. Althagafi et al.^37^ assessed the correlation between aesthetic perception and varying educational levels among dental students, revealing that 5th- year dental students had a higher level of aesthetic perception than 4th- year students. Despite the study being conducted among individuals with an undergraduate education, it was revealed that the elevation in education level showed a linear correlation with the enhancement of knowledge and awareness about aesthetic perception.

Kovačić et al.^38^ found that students with advanced education who underwent clinical internship training exhibited a more consistent and critical perspective in aesthetic evaluations, indicating that enhanced perceptual skills were developed through the accumulation of knowledge. In our study evaluating the impact of education on aesthetic perception, it was determined that participants with postgraduate education exhibited a considerably greater rate of selecting the proper aesthetic smile compared to other groups (*p* < 0.05). However, the relatively high educational level of the layperson group may have influenced aesthetic judgements and should be considered when interpreting these findings. Although the observed differences between evaluator groups were statistically significant, their clinical relevance lies in the divergent aesthetic priorities revealed. Professionals prioritized gingival display and proportionality, while laypersons often emphasised tooth visibility and brightness. These findings highlight the importance of patient-professional communication in treatment planning to align aesthetic expectations.

Consequently, our study revealed the significance of professional competence and educational attainment on smile aesthetics. Perceptions of smiles among dentists and persons with postgraduate degrees were more critical. The results demonstrated that, in aesthetic dentistry applications, it is essential to take into account patients’ personal expectations and their educational background.

This study presents several limitations that warrant consideration. First, due to the retrospective design, the sample size was limited to available patient records that met the strict inclusion and standardisation criteria, which may restrict the generalisability of the findings. Second, smile esthetics was assessed using two-dimensional static photographs; therefore, dynamic aspects of smiling, such as lip mobility and spontaneous facial expressions, were not captured. Third, the relatively high educational level of the layperson group may have influenced aesthetic judgements and reduced the variability of the sample, potentially affecting the external validity of the results. Finally, cultural and regional factors may also have influenced aesthetic perceptions; thus, the findings may not be universally applicable. Future studies with larger and more diverse populations, balanced professional subgroups, and dynamic smile assessments are warranted.

## Conclusions

In the design of smile aesthetics, conventional aesthetic standards may not always align with patient expectations. The present study demonstrated that aesthetic perceptions differ between dental professionals and laypersons, with distinct evaluative priorities. However, it should be acknowledged that the Smile Index represents only one quantitative and static dimension of smile evaluation and may not fully capture the overall complexity and dynamic nature of smile esthetics. Therefore, in addition to objective parameters, smile design should be individualised, and patient expectations should be carefully considered during treatment planning.

Future studies incorporating dynamic smile analysis and multiple esthetic parameters may provide a more comprehensive understanding of smile perception.

## Data Availability

All data generated or analysed during this study are included in this article.
